# Evaluation of artificial intelligence–based software programs applied to the radiographic and photographic identification of dental implant systems

**DOI:** 10.1111/jopr.70155

**Published:** 2026-04-22

**Authors:** Sohil A. Kazim, Charles J. Goodacre

**Affiliations:** ^1^ Advanced Education Program in Implant Dentistry Loma Linda University School of Dentistry Loma Linda California USA; ^2^ Department of Restorative Dentistry Advanced Specialty Education Program in Prosthodontics Rutgers School of Dental Medicine Newark New Jersey USA

**Keywords:** artificial intelligence in dentistry, dental implant identification, radiographic and photographic analysis

## Abstract

**Purpose:**

The purpose of this evaluation study was to assess the performance of ChatGPT‐5, Google Gemini (v2.5), and Microsoft Copilot in identifying five demonstration dental implant systems and four implants from real clinical cases based on clinical and radiographic images.

**Materials and Methods:**

Clinical photographs of the demonstration implant platforms and corresponding radiographs of NobelActive (NobelActive; Nobel Biocare), NobelParallel Conical Connection (NobelParallel CC; Nobel Biocare), NobelReplace Conical Connection (NobelReplace CC; Nobel Biocare), Straumann Bone Level Conical (Straumann BLC; Institut Straumann), and Straumann BLX (Straumann BLX; Institut Straumann), were entered into ChatGPT‐5, Google Gemini (v2.5), and Microsoft Copilot with one standardized prompt. In addition, four previously placed implants from real patient cases (NobelActive, NobelReplace CC, Straumann Bone Level Tapered [BLT], and Straumann BLX) were analyzed using the same software programs and standardized prompt. All inputs were conducted in a clean session. All responses from ChatGPT‐5, Gemini, and Copilot software programs were compared for accuracy and descriptive completeness.

**Results:**

ChatGPT‐5 correctly identified two of the five demonstration implant systems, but did not definitively identify any of the four real clinical cases. Google Gemini failed to correctly identify any implant system in either the demonstration or clinical cases. Microsoft Copilot did not correctly identify the demonstration implants but correctly identified one of the four real clinical cases (Straumann BLX). Diagnostic overlap among tapered internal‐connection systems was a common source of misclassification.

**Conclusions:**

None of the evaluated artificial intelligence (AI) software programs consistently identified dental implant systems across both demonstration and real clinical cases. Although ChatGPT‐5 showed partial diagnostic capability in demonstration implants and Microsoft Copilot correctly identified one real clinical case, overall system‐level identification remained unreliable.

The accurate identification of dental implant systems is an essential step in restorative and maintenance procedures for partially dentate and edentulous patients. When patients present for prosthodontic treatment without documentation of the previously placed implant, clinicians must rely on visual inspection and radiographic analysis to determine the implant type and compatible restorative components.^1^, ^2^, ^3^ Errors in implant identification may lead to inappropriate abutment selection, prosthesis misfit, or mechanical complications.^4^, ^5^, ^6^

Clinicians frequently encounter patients with implants placed many years earlier for which surgical or prosthetic documentation is unavailable. In such situations, accurate implant identification becomes critical for selecting compatible restorative components, performing maintenance procedures, or managing complications. However, implant identification can be challenging because many implant systems share similar macrodesign features, thread configurations, and connection geometries. In addition, implants placed decades ago may no longer be actively marketed or may have undergone design modifications, further complicating recognition. Consequently, clinicians often rely on time‐consuming comparison with reference libraries or consultation with manufacturers, and accurate identification remains uncertain in many cases.

More than 1500 implant systems are currently available worldwide, many of which share similar macro‐ and microdesigns, internal connection geometries, and surface characteristics.^7^, ^8^, ^9^ The resulting overlap among systems complicates the diagnostic process, even for experienced clinicians. Traditional approaches to identifying unknown implants include radiographic comparison with reference libraries, use of online databases such as WhatImplantisThat.com, and consultation with manufacturers or colleagues.^10^, ^11^, ^12^ Several national and institutional dental implant registries have been established in countries such as Sweden, Finland, the United States, and Canada; however, they primarily record newly placed implants and lack comprehensive data regarding previously placed implants.^13^ Although these methods can be effective, they are often time‐consuming, dependent on clinician experience, and limited by incomplete radiographic or photographic documentation.^14^

Several dedicated dental software platforms, including Spotimplant, Implantif.AI, SearchImplant, and WhatImplantIsThat.com, have been specifically developed to support implant identification through structured radiographic comparison and searchable databases. Most of these systems operate on subscription or pay‐per‐use models and are designed exclusively for implant recognition tasks. In parallel, artificial intelligence (AI) has emerged as a powerful adjunctive tool in dentistry, assisting with radiographic interpretation, diagnosis, and clinical decision‐making.^15^, ^16^, ^17^ Recent advancements in generative AI and large language models (LLMs) have enabled the processing of both textual and visual data.^18^, ^19^ Platforms such as ChatGPT‐5 (OpenAI), Google Gemini (Alphabet Inc.), and Microsoft Copilot (Microsoft Corp.) can analyze descriptive prompts alongside radiographic and photographic features, potentially offering a novel pathway for dental implant system identification. Among these systems, ChatGPT is the most widely adopted AI platform as of August 2025, with approximately 700 million weekly users worldwide.^20^ ChatGPT was unveiled in November 2022 as a digital information resource to address inquiries on a variety of scientific subjects.^20^ Its vast database, which has been compiled from books, articles, and websites, imitates statistical patterns of language using an LLM.^21^ The architecture of ChatGPT has been reported to create responses based on the text input by using a neural network.^22^ OpenAI launched ChatGPT‐4 in March 2023, an improved version intended to respond to complex queries more efficiently.^23^, ^24^ More recently, ChatGPT‐5, which requires a fee, was released in August 2025. Microsoft introduced Copilot in 2023 and soon after was embedded in Microsoft 365 apps, including Word, Excel, and PowerPoint, and then in Edge on the browser sidebar. In 2024, it became available on iOS and Android. Gemini was launched in December 2023 as an advanced replacement of Bard, which had been launched earlier that year. Its initial version (1.0) was subsequently improved, and version 2.5 was released in the spring of 2025. As with ChatGPT, there are tiered options.

Because of the increasing clinical use of AI software programs such as ChatGPT, Google Gemini, and Microsoft Copilot, this study was conducted to evaluate their reliability and diagnostic performance in identifying dental implant systems based on clinical photographs of demonstration implant platforms and corresponding periapical radiographs. In addition to the demonstration implants, real patient cases were incorporated to enhance clinical applicability. The purpose of this study was to evaluate and compare the ability of three AI‐based software programs (ChatGPT‐5, Google Gemini [v2.5], and Microsoft Copilot) to identify dental implant systems under standardized conditions and in real clinical scenarios. The research hypothesis was that ChatGPT‐5, Google Gemini, and Microsoft Copilot could accurately identify different dental implant systems when provided with clinical photographs of the implant platform and corresponding periapical radiographs.

## MATERIALS AND METHODS

Five commercially available demonstration implant systems were selected for controlled evaluation: NobelActive (NobelActive; Nobel Biocare), NobelParallel Conical Connection (NobelParallel CC; Nobel Biocare), NobelReplace Conical Connection (NobelReplace CC; Nobel Biocare), Straumann Bone Level Conical (Straumann BLC; Institut Straumann), and Straumann BLX (Straumann BLX; Institut Straumann). All demonstration implants were supplied directly by the respective manufacturers.

For each demonstration implant, one clinical photograph of the implant platform and one corresponding periapical radiograph were obtained. Clinical photographs were captured using a mirrorless digital camera (Z5; Nikon Corp.) with a macro lens (NIKKOR Z MC 105 mm f/2.8 VR S; Nikon Corp.) under diffused illumination to minimize reflections and shadows (Figure 1a–e). Periapical radiographs were acquired using a paralleling technique (ProX; Planmeca Oy) at 70 kVp and 8 mA with an exposure time of 0.16 s (Figure 2a–e). All photographs and radiographs were exported and saved in JPEG format without additional enhancement or editing.

**FIGURE 1:**
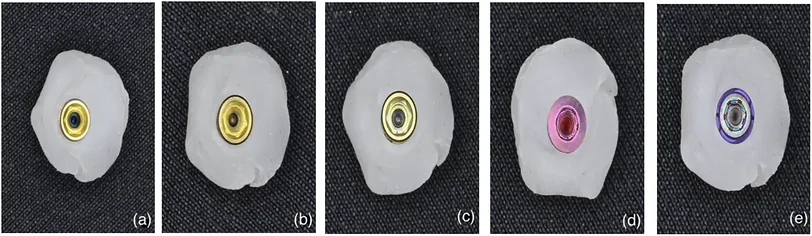
Photographs of the included demonstration implant platforms. (a) NobelActive (NobelActive; Nobel Biocare). (b) NobelParallel Conical Connection (NobelParallel CC; Nobel Biocare). (c) NobelReplace Conical Connection (NobelReplace CC; Nobel Biocare). (d) Straumann Bone Level Conical (Straumann BLC; Institut Straumann). (e) Straumann BLX (Straumann BLX; Institut Straumann).

**FIGURE 2:**
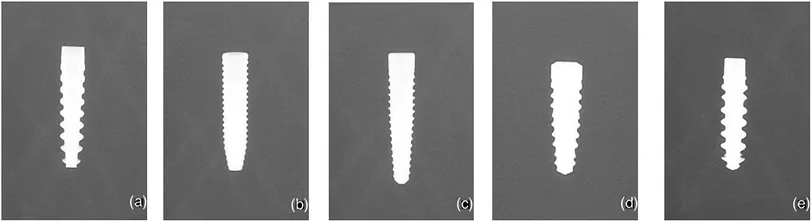
Radiographs of the included demonstration implant bodies. (a) NobelActive (NobelActive; Nobel Biocare). (b) NobelParallel Conical Connection (NobelParallel CC; Nobel Biocare). (c) NobelReplace Conical Connection (NobelReplace CC; Nobel Biocare). (d) Straumann Bone Level Conical (Straumann BLC; Institut Straumann). (e) Straumann BLX (Straumann BLX; Institut Straumann).

To enhance clinical applicability, four previously placed implants from real patient cases were included. These implants were retrospectively verified and consisted of NobelActive (NobelActive; Nobel Biocare), NobelReplace Conical Connection (NobelReplace CC; Nobel Biocare), Straumann Bone Level Tapered (Straumann BLT; Institut Straumann), and Straumann BLX (Straumann BLX; Institut Straumann). For each case, one intraoral clinical photograph of the implant platform (Figure 3a–d) and one corresponding periapical radiograph were obtained from existing clinical records (Figure 4a–d). No additional image manipulation was performed. The use of retrospective clinical images was conducted in accordance with institutional ethical standards, and all patient identifiers were removed before analysis.

**FIGURE 3:**
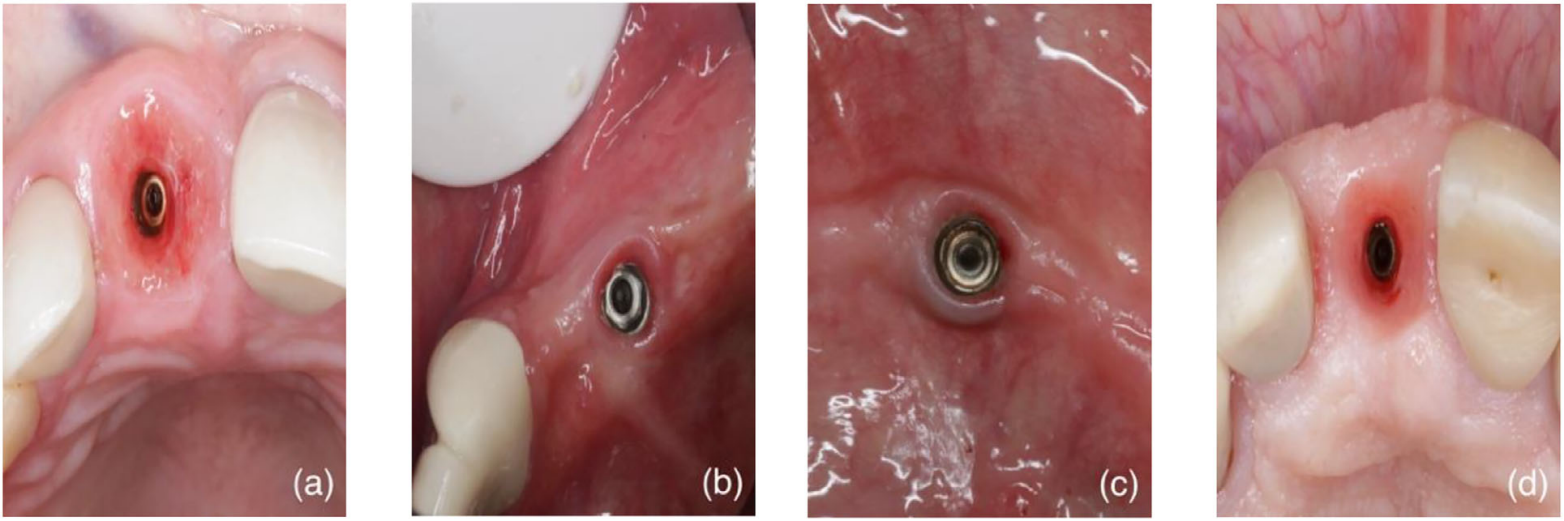
Photographs of the included implant platforms of real cases. (a) NobelActive (NobelActive; Nobel Biocare). (b) NobelReplace Conical Connection (NobelReplace CC; Nobel Biocare). (c) Straumann Bone Level Tapered (Straumann BLT; Institut Straumann). (d) Straumann BLX (Straumann BLX; Institut Straumann).

**FIGURE 4:**
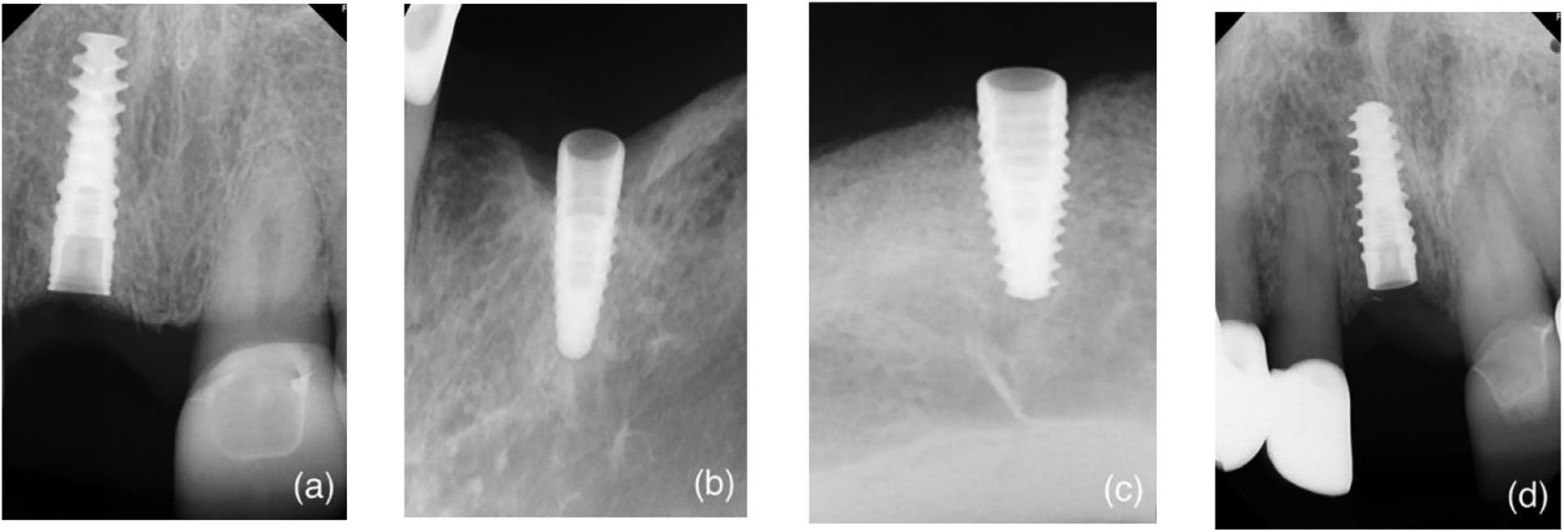
Radiographs of the included implant bodies of real cases. (a) NobelActive (NobelActive; Nobel Biocare). (b) NobelReplace Conical Connection (NobelReplace CC; Nobel Biocare). (c) Straumann Bone Level Tapered (Straumann BLT; Institut Straumann). (d) Straumann BLX (Straumann BLX; Institut Straumann).

Each implant (five demonstration implants and four real clinical cases; total *n* = 9) was entered separately into three AI‐based software programs: ChatGPT‐5 (OpenAI), Google Gemini (v2.5; Alphabet Inc.), and Microsoft Copilot (Microsoft Corp.). All evaluations were conducted in clean sessions without prior contextual prompts to minimize bias. The identical standardized prompt was used for all inputs: “Identify the dental implant system and manufacturer based on the attached clinical photograph and corresponding periapical radiograph.” A simplified zero‐shot prompt was intentionally used to simulate a real‐world clinical scenario in which a clinician might request implant identification without extensive prompt engineering or iterative contextual refinement. This approach allowed standardized comparison among the evaluated AI platforms while minimizing bias introduced by complex prompt optimization. Repeated trials were not performed because the study design aimed to replicate a single‐query clinical scenario; however, it is acknowledged that AI responses may vary across repeated inputs, representing a limitation of the study. Responses were recorded and evaluated for system‐level accuracy. Each implant case was assigned a unique identifier prior to analysis to ensure consistent tracking of AI outputs. The responses generated by each AI platform were independently matched to the corresponding verified implant system for accuracy assessment. A response was considered correct only if the specific implant system and manufacturer matched the verified implant. Broad differentials, manufacturer‐only identifications without specific system designation, or multiple‐system listings were categorized as incorrect for accuracy calculation purposes. Descriptive interpretation of implant features (e.g., macrodesign, thread morphology, and platform characteristics) was also qualitatively reviewed to assess feature‐recognition capability. Because no previous investigations have evaluated multimodal generative AI software programs for dental implant system identification, a formal power analysis was not feasible. Therefore, this study was designed as an exploratory pilot investigation intended to provide preliminary observations regarding the diagnostic capabilities of currently available generative AI platforms.

## RESULTS

The research hypothesis was rejected because none of the evaluated AI software programs consistently achieved accurate implant system‐level identification across both demonstration and real clinical cases.

In the five controlled demonstration implants, ChatGPT‐5 correctly identified two systems (NobelReplace Conical Connection and Straumann BLX). In the remaining demonstration cases, it misclassified the implants despite appropriately describing several relevant macrodesign and general implant morphology. Google Gemini did not correctly identify any demonstration implant and repeatedly attributed them to an incorrect system, regardless of variations in macrodesign and connection geometry. Microsoft Copilot also did not achieve definitive system‐level identification in any of the demonstration implants, although it frequently generated broad differentials that included multiple manufacturers (Table 1).

**TABLE 1:** Comparison of responses from ChatGPT‐5, Google Gemini, and Microsoft Copilot in identifying five demonstration dental implant systems.

Implant system	Key diagnostic features	ChatGPT results	Google Gemini results	Microsoft Copilot results
NobelActive	Tapered body, flared coronal portion, aggressive self‐cutting apical threads, internal conical hex	Incorrect. Identified as Straumann BLT; recognized taper and thread form but failed to associate aggressive apical geometry and flared coronal profile with NobelActive	Incorrect. Classified as NobelReplace Select, interpreted as straight‐walled with tri‐channel interface	Broad differential (Straumann BLT or Nobel CC or Zimmer TSV); recognized tapered geometry but no definitive identification
NobelParallel Conical Connection (CC)	Parallel‐walled macrodesign, uniform threads, flat coronal shoulder, internal conical hex	Incorrect. Misidentified as NobelReplace CC; did not distinguish parallel‐walled macrodesign from tapered configuration	Incorrect. Classified as NobelReplace Select, interpreted as straight‐walled tri‐channel system	Suggested Zimmer or Nobel or BioHorizons tapered systems; failed to recognize parallel‐wall macrodesign
NobelReplace Conical Connection (CC)	Tapered macro‐geometry, uniform V‐threads, gold platform, internal conical hex	Correct. Recognized NobelReplace CC based on tapered body, platform color, and uniform threads	Incorrect. Classified as NobelReplace Select	Listed BioHorizons or Zimmer TSV or MIS; described tapered conical connection but no correct system recognition
Straumann Bone Level Conical (BLC)	Pink CrossFit platform, moderate taper, short machined collar, symmetrical threads	Incorrect. Misidentified as Straumann BLT; recognized CrossFit‐type morphology but could not distinguish BLC from BLT	Incorrect. Classified as NobelReplace Select	Listed Nobel CC or Straumann BLT or BioHorizons; recognized internal conical taper but no accurate system assignment
Straumann BLX	Fully tapered body‐active design, variable thread depth or pitch, purple CrossFit platform	Correct. Identified as Straumann BLX; correctly noted aggressive macrogeometry and platform characteristics	Incorrect. Classified as NobelReplace Select	Identified as Straumann bone‐level type; did not recognize BLX aggressive macrodesign

In the four real clinical cases, overall identification performance declined further. ChatGPT‐5 did not correctly identify any of the real clinical implants and commonly generated multiple possible systems when confronted with overlapping tapered internal‐connection features. Google Gemini again failed to provide a correct identification and most frequently categorized implants as Zimmer Tapered Screw‐Vent–type systems despite inconsistent radiographic morphology. Microsoft Copilot correctly identified one real clinical implant (Straumann BLX), whereas the remaining cases were assigned to generalized system categories without definitive confirmation (Table 2).

**TABLE 2:** Comparison of responses from ChatGPT‐5, Google Gemini, and Microsoft Copilot in identifying four dental implants from real clinical cases.

Implant system	Key diagnostic features	ChatGPT results	Google Gemini results	Microsoft Copilot results
NobelActive	Tapered body, flared coronal portion, aggressive self‐cutting apical threads, internal conical hex	Incorrect. Generated differential, including NobelActive and BioHorizons Tapered Internal; no definitive implant system‐level identification	Incorrect. Identified as Zimmer Tapered Screw‐Vent (TSV); emphasized internal hex and triple‐lead threads inconsistent with verified system	Incorrect. Provided narrowed differential, including NobelActive but listed multiple possible systems without definitive identification
NobelReplace Conical Connection (CC)	Tapered macro‐geometry, uniform V‐threads, gold platform, internal conical hex	Incorrect. Identified as Zimmer Tapered Screw‐Vent; focused on tapered geometry and internal hex features	Incorrect. Identified as Zimmer Tapered Screw‐Vent; did not distinguish NobelReplace macrodesign characteristics	Incorrect. Included NobelReplace among several possible systems (Astra, Straumann Bone Level) but did not provide definitive identification
Straumann Bone Level Tapered (BLT)	Tapered body, symmetrical threads, internal conical CrossFit connection, bone‐level placement	Incorrect. Identified as Astra Tech OsseoSpeed EV; emphasized conical connection and parallel‐walled morphology	Incorrect. Identified as Zimmer Tapered Screw‐Vent; cited internal hex and cylindrical body design	Incorrect. Generated differential, including Straumann BLT but did not assign definitive implant system‐level identification
Straumann BLX	Fully tapered body‐active design, variable thread depth or pitch, purple CrossFit platform	Incorrect. Identified as NobelActive based on aggressive thread configuration	Incorrect. Identified as Astra Tech OsseoSpeed EV; emphasized conical connection and platform coloration	Correct. Identified as Straumann BLX based on aggressive tapered macrodesign and characteristic internal connection features

When all nine implants were considered collectively, ChatGPT‐5 achieved the highest number of correct identifications, followed by Microsoft Copilot; Google Gemini did not provide a correct system‐level identification in any case. Across programs, misclassification most commonly occurred among tapered internal‐connection systems, particularly between NobelActive and Straumann BLX designs and between NobelReplace and Zimmer Tapered Screw‐Vent–type systems. Although descriptive recognition of implant features was frequently appropriate, translation of these observations into accurate and definitive implant system identification remained inconsistent.

## DISCUSSION

This study evaluated the diagnostic performance of three AI software programs—ChatGPT‐5, Google Gemini (v2.5), and Microsoft Copilot—in identifying dental implant systems using clinical photographs and corresponding periapical radiographs. Although partial feature recognition was frequently observed, none of the programs consistently achieved accurate implant system‐level identification across both demonstration implants and real clinical cases.

ChatGPT‐5 demonstrated the strongest overall performance, correctly identifying two of the five demonstration implants. In these cases, the software appropriately described key macrodesign features, including body taper, thread configuration, and platform characteristics. However, when presented with real clinical cases, ChatGPT‐5 did not achieve definitive identification. The reduction in performance suggests that factors commonly present in clinical scenarios—such as soft tissue coverage and radiographic angulation variability—may significantly affect AI interpretation. The findings indicate that while AI software programs can recognize descriptive implant features, consistent translation of those features into accurate system‐level identification remains limited. Google Gemini did not correctly identify any implant system in either the demonstration or real clinical cases. Responses frequently defaulted to a single implant family despite clear morphological differences among systems. Microsoft Copilot demonstrated limited but notable performance by correctly identifying one real clinical implant (Straumann BLX). In several additional cases, Copilot generated narrowed differentials that included appropriate manufacturers, indicating partial feature recognition. However, definitive identification was not consistently achieved.

Across all programs, misclassification most frequently occurred among tapered internal‐connection systems. Diagnostic overlap was particularly evident between NobelActive and Straumann BLX designs and between NobelReplace and Zimmer Tapered Screw‐Vent–type systems. These systems share similarities in body taper, thread configuration, and overall implant morphology, which likely contribute to confusion when interpreted by language‐based multimodal models. The findings suggest that current AI software programs rely heavily on generalized pattern associations rather than structured manufacturer‐specific implant databases. Although a formal confusion matrix analysis was not performed, recurring patterns of misclassification were qualitatively identified among morphologically similar implant systems. Generative AI systems are also susceptible to a phenomenon commonly referred to as “hallucination,” in which models generate confident but incorrect responses due to probabilistic language prediction rather than true domain‐specific understanding. This limitation may partly explain the incorrect implant classifications observed in this study, particularly when multiple implant systems share overlapping morphological characteristics.

To the authors’ knowledge, this is the first investigation to evaluate AI‐driven implant system identification. Recent investigations evaluating chatbot responses in prosthodontics and restorative dentistry have similarly reported variable accuracy and inconsistent reasoning patterns when answering domain‐specific questions.^25^ Previous studies have reported the use of conventional image‐based databases such as WhatImplantIsThat.com or machine‐learning models trained on specific radiographic datasets for implant recognition, showing variable accuracy depending on dataset size and implant diversity.^1^, ^3^, ^11^ However, no published research has compared the capability of multimodal generative AI software programs to identify dental implant systems. The present findings align with previous reviews, indicating that non‐domain‐specific LLMs perform poorly in specialized diagnostic contexts without targeted dataset training.^17^, ^18^, ^19^ Similar limitations in AI performance have also been observed in dental education and examination settings.^26^

The primary objective of this exploratory study was to assess system‐level identification accuracy rather than to perform detailed quantitative modeling; therefore, descriptive analysis was considered appropriate for the study design. This study has several limitations. The number of evaluated implant systems was limited, and all implants were derived from two manufacturers. The selection of implant systems and clinical cases was based on availability and, therefore, may represent convenience sampling, which could introduce selection bias. The evaluation was based on a single standardized prompt per case, and software performance may vary with alternative phrasing. Additionally, the rapidly evolving nature of AI software programs may influence future performance. Nonetheless, inclusion of real clinical cases strengthens the applicability of the findings and demonstrates that performance in controlled demonstration settings does not necessarily translate to real‐world conditions. In addition, the demonstration implant radiographs were obtained without surrounding bone simulation because they were acquired from isolated implant samples provided by manufacturers. This imaging condition may have influenced radiographic contrast and the visibility of certain implant features compared with real clinical situations. However, the inclusion of radiographs from real patient cases partially mitigates this limitation and improves the clinical relevance of the findings. No comparison with human expert performance was included; therefore, the relative diagnostic performance of the evaluated AI systems compared with clinicians could not be assessed.

Future research should focus on the development of implant‐specific AI models trained on curated radiographic datasets representing diverse implant systems and clinical scenarios. Integration of structured manufacturer databases and validated image libraries may enhance diagnostic precision. Until such systems are available, AI software programs should be regarded as adjunctive tools that may assist in narrowing differential possibilities but cannot replace clinician expertise, component verification, or manufacturer confirmation.

## CONCLUSIONS

Within the limitations of this exploratory in silico study, none of the evaluated AI software programs consistently identified dental implant systems across both demonstration implants and real clinical cases. ChatGPT‐5 demonstrated the strongest overall performance by correctly identifying two controlled demonstration implants, whereas Microsoft Copilot correctly identified one real clinical implant. Google Gemini did not achieve system‐level identification in any case.

Although the software programs frequently recognized general implant features, accurate and reliable translation of these observations into definitive implant system identification remained inconsistent. Factors such as prompt structure, image characteristics, and overlapping implant morphologies may influence diagnostic performance. At present, generative AI software programs may assist in narrowing differential possibilities but should not replace clinical judgment, radiographic interpretation, or manufacturer verification when managing patients with undocumented implants.

## CONFLICT OF INTEREST STATEMENT

The authors declare no conflicts of interest.

## Data Availability

Data sharing is not applicable to this article as no datasets were generated or analyzed during the current study.

## References

[1] Sannino G , Bollero P , Barlattani Jr A , Bollero R . Implant identification by radiographic analysis: a systematic review. J Prosthet Dent. 2020;123:879‐886.

[2] Resnik RR , Misch CE . Diagnostic imaging in implant dentistry. In: Misch CE , editor. Dental implant prosthetics. 3rd ed. St Louis, MO: Elsevier; 2025.

[3] Lim J , Kang DW , Kim Y , Kim S . A web‐based implant recognition system using radiographic morphology. Clin Oral Implants Res. 2019;30:110‐117.

[4] Goodacre CJ , Bernal G , Rungcharassaeng K , Kan JYK . Clinical complications with implants and implant prostheses. J Prosthet Dent. 2003;90:121‐132. 10.1016/S0022-3913(03)00212-9 12886205

[5] Wittneben JG , Buser D , Salvi GE , Bürgin W , Hicklin S , Brägger U . Complications and maintenance of implant prostheses. Int J Prosthodont. 2020;33:467‐478.

[6] Borges GA , Lima E , Almeida J , Carvalho A , Naves LZ , Prado CJ . Misfit of implant prostheses: causes and consequences. J Prosthet Dent. 2022;128:457‐465.

[7] Jivraj S , Chee W , Corrado P . Implant system variability and prosthodontic implications. Br Dent J. 2017;223:401‐409.

[8] Alqutaibi AY , Algabri RS , Sailer I , Sailer R . Morphologic diversity among implant macrodesigns. Int J Oral Maxillofac Implants. 2021;36:375‐383.

[9] Huang C , Zhang X , Liu J , Li D , Zhang Y . Comparison of implant connection types and mechanical performance. J Mech Behav Biomed Mater. 2020;110:103886.

[10] Howell K , Farley N . WhatImplantIsThat.com: radiographic implant database. Revive Dental Implant Center. 2025 Nov.

[11] Bidra AS , Rungruanganunt P , Rungcharassaeng K , Goodacre CJ . Contemporary approaches to implant identification. J Prosthet Dent. 2021;125:246‐252.

[12] Buser D , Chappuis V , Bornstein MM , Wittneben JG , Frei M , Belser UC . Clinical considerations in implant selection. Periodontol 2000. 2019;81:194‐208.

[13] Naemi R , Barikani HR , Shahmoradi L . Dental implant quality registries and databases: a systematic review. J Educ Health Promot. 2021;10:214. 10.4103/jehp.jehp_1302_20 PMC8318161 34395651

[14] Al‐Johany SS , Al‐Amri MD , Al‐Yami SM , Al‐Ahdal K . Diagnostic challenges with undocumented implants. Clin Implant Dent Relat Res. 2019;21:847‐854.

[15] Schwendicke F , Samek W , Krois J . Artificial intelligence in dentistry: current applications and future directions. J Dent Res. 2023;102:13‐22.

[16] Revilla‐León M , Gómez‐Polo M , Özcan M . Artificial intelligence in prosthodontics: a systematic review. J Prosthet Dent. 2023;130:387‐396. 10.1016/j.prosdent.2021.06.001 34281697

[17] Wismeijer D , Revilla‐León M , Roca‐Monzo J , Goodacre CJ . Digital and AI‐assisted prosthodontics. J Prosthet Dent. 2024;131:214‐221.

[18] Schwendicke F , Samek W , Krois J . Artificial intelligence in dentistry: chances and challenges. J Dent Res. 2020;99:769‐774. 10.1177/0022034520915714 PMC7309354 32315260

[19] Liu Z , Zhang Y , Wu B , et al. Performance of large language models in dentomaxillofacial radiology: a systematic review. Dentomaxillofac Radiol. 2024;53. 10.1093/dmfr/twaf060 PMC12653761 40796316

[20] Macdonald C , Adeloye D , Sheikh A , Rudan I . Can ChatGPT draft a research article? An example of population‐level vaccine effectiveness analysis. J Glob Health. 2023;13:01003. 10.7189/jogh.13.01003 PMC9936200 36798998

[21] Stokel‐Walker C . ChatGPT listed as author on research papers: many scientists disapprove. Nature. 2023;613:620‐621. 10.1038/d41586-023-00107-z 36653617

[22] Salvagno M , Taccone FS , Gerli AG . Can artificial intelligence help for scientific writing? Crit Care. 2023;27:75. 10.1186/s13054-023-04380-2 PMC9960412 36841840

[23] Abdullahi T , Singh R , Eickhoff C . Learning to make rare and complex diagnoses with generative AI assistance: qualitative study of popular large language models. JMIR Med Educ. 2024;10:e51391. 10.2196/51391 PMC10900078 38349725

[24] Wang YM , Chen TJ . ChatGPT surges ahead: GPT‐4 has arrived in the arena of medical research. J Chin Med Assoc. 2023;86:784‐785. 10.1097/JCMA.0000000000000955 PMC12755478 37406215

[25] Chau RCW , Thu KM , Yu OY , Hsung RT‐C , Wang DCP , Man MWH , et al. Evaluation of chatbot responses to text‐based multiple‐choice questions in prosthodontic and restorative dentistry. Dent J (Basel). 2025;13(7):279. 10.3390/dj13070279 PMC12293279 40710124

[26] Chau RCW , Thu KM , Yu OY , Hsung RTC , Lo ECM , Lam WYH . Performance of generative artificial intelligence in dental licensing examinations. Int Dent J. 2024;74:616‐621. 10.1016/j.identj.2023.12.007 PMC11123518 38242810

